# Resolutions on the Death of Dr. Gilman P. Robinson, Presented to the Atlanta Society of Medicine

**Published:** 1902-11

**Authors:** V. O. Hardon, J. C. Olmsted, Bernard Wolff


### RESOLUTIONS ON THE DEATH OF DR. GILMAN P. ROBINSON, PRESENTED TO THE ATLANTA SOCIETY OF MEDICINE.

WHEREAS, In the dispensation of Providence, death has removed from among us Dr. Gilman P. Robinson, a member of this association and an honored member of the medical profession of the city of Atlanta; therefore, be it

*Resolved,* That in the death of Dr. Robinson this society has lost a member who represented to a high degree the culture and ability which should render our profession worthy of respect and confidence, and which should elevate it to the plane which a learned profession should occupy in the esteem of the public.

*Resolved,* That we deplore the loss of a worthy member who, but for the hindrances placed in his path by ill-health, would have occupied a worthy place in this society and in the medical profession of this city.

*Resolved,* That this society extends to the bereaved wife of the deceased its fullest sympathy and commiseration in this hour of her deep sorrow.

*Resolved,* That these resolutions be published in THE ATLANTA JOURNAL-RECORD OF MEDICINE and a copy be sent to the bereaved wife of our late associate.

V. O. HARDON,
J. C. OLMSTED,
BERNARD WOLFF

THE Tri-State Medical Society of Alabama, Georgia and Tennessee met in Birmingham, October 7, 8 and 9. There were about one hundred and fifty members present. The papers were numerous and meritorious, and were freely discussed.

The Atlanta members who attended the meeting were enthusiastic in their praise of the cordial welcome and pleasant treatment accorded them by the profession at Birmingham. Much of the success of the social side of the gathering was due to the efforts of Dr. Geo. Stubbs, well remembered as a former resident of Atlanta, and of Dr. R. C. Bankston. The officers elected for the ensuing year are: Dr. Michael Hoke, of Atlanta, President; Dr. Lewis C. Morris, of Birmingham, First Vice-President; Dr. W. L. Nolan, of Chattanooga, Second Vice-President; Dr. Pete, of Georgia, Third Vice-President. The next meeting will be held at Atlanta, October, 1903, when we hope to have the opportunity of entertaining the society in a manner befitting.

---

DR. DAVID CERNA, formerly of Texas, but now of Monclova, Mexico, has sent us copies of a newspaper, *El Espectador*, published at Monterey, Mexico.

They contain a history of the discovery of anesthesia by Crawford W. Long, written by Dr. Cerna. He is an enthusiastic advocate of the claims of Dr. Long, and refers to the action of the Georgia Legislature in according a place for the bust of Dr. Long in the Hall of Fame as a great triumph for truth and justice. Dr. Cerna refers to the late Dr. Luther B. Grandy as one of the great champions in the great controversy, and whose honest efforts have finally been crowned, as they deserve to be, with great success.

